# Stabilization of the critically ill neonate awaiting transport

**DOI:** 10.1186/1824-7288-41-S1-A15

**Published:** 2015-09-24

**Authors:** Maurizio Gente, Domenico Di Lallo, Francesco Franco, Roberto Aufieri, Piermichele Paolillo, Mario De Curtis

**Affiliations:** 1 Department of Pediatrics and Infant Neuropsychiatry, Neonatal Emergency Transport Service, Sapienza University of Rome, Rome, Italy; 2 Regional Health Department of Lazio, Rome, Italy; 3 Division of Neonatology and Neonatal Intensive Care, Casilino General Hospital, Rome, Italy; 4 University of Rome “Sapienza”, Department ofPaediatrics and Infant Neuropsychiatry, Rome, Italy

## Introduction

An appropriate stabilization before transport is essential to reduce adverse events [1-5]. The aim of this study was to describe the characteristics of a cohort of newborns transported and to evaluate the association between stabilization time and change of Transport Risk Index of Physiologic Stability (TRIPS score)[6].

## Materials and methods

The database of the Neonatal Emergency TransportService in Lazio Region andall newborns transported within May 2009-December 2012 wereanalyzed (N=2,331). A multinomial logistic regression model was used to study the association between stabilization time and improvement and deterioration in TRIPS score in reference to no change, adjusting for potential confounders. Mortality Index for Neonatal Transportation score(MINT) [7] was analysed but not included in the multivariate model due to the covariation with the TRIPS score. In order to evaluate the potential interaction with stabilization times, the data analysis was stratified by perinatal level of care (I, II, III). Two-tailed p-values were considered at 5% significance level. 

## Results

**Figure 1:**
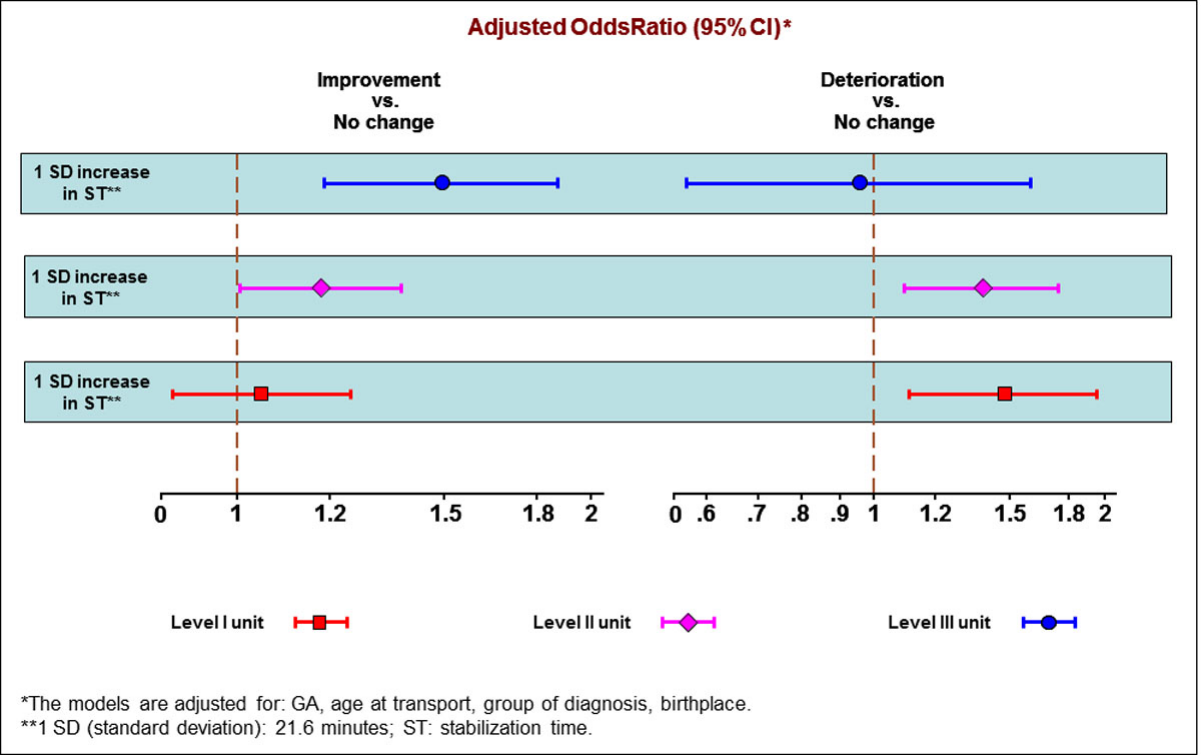
Association between stabilization time and Transport Risk Index of Physiologic Stability change: results of adjusted multinomial logistic regression models stratified by birth centre level. Lazio, May 2009-December 2012.

Table 1 shows descriptive statistics of transport characteristics by birth centre level. Median GA was 36 weeks and 6.6% had less than 28 weeks. Median age at transport was 4.9 hours. The most frequent diagnosis was respiratory diseases in all birth centre levels (52% overall). Mean MINT score was 3.1 and increased from 1.4 to 5.4 across the three levels of care. Median stabilization times were 25 minutes in level I and III, and 30 in level II. Overall, median pre-transport TRIPS score was 6; the highest mean value was observed in level III units (11.7). Overall, 72.9% of all infants showed no TRIPS score change, 22.7% an improvement, and 4.4% a deterioration (4.9% in level III). Figure 1 shows the results from multinomial regression analysis of improvement and deterioration in TRIPS score in reference to no change. An association between stabilization time and TRIPS change was observed, depending on the level of the centre: an increase in stabilization time was associated with increased odds of deterioration (+48% for 1 SD increase, 21.6 minutes) in level I; by contrast, an increase in stabilization time was associated with increased odds of improvement (+49%) in level III. Both effects were observed in level II units. 

**Table 1:** Infants characteristics byperinatal level of care. Lazio, 2009-December 2012

	Birth centre level			
	I N=966	II N=651	III N=714	Total N=2331
**Gestational age (wks)**				
22-27 (%)	8 (0.8)	30 (4.6)	117 (16.4)	155 (6.6)
28-31 (%)	37 (3.8)	52 (8.0)	212 (29.7)	301 (12.9)
32-36 (%)	313 (32.4)	200 (30.7)	280 (39.2)	793 (34.0)
37+ (%)	608 (62.9)	369 (56.7)	105 (14.7)	1082 (46.4)
Mean (SD)	37.0 (2.8)	36.1 (3.9)	31.9 (4.1)	35.2 (4.2)
Median	37	37	32	36
**Age at transport (hours)**				
Mean (SD)	15.2 (16.8)	15.0 (16.1)	6.8 (11.2)	12.6 (15.6)
Median	6.6	6.9	3.1	4.9
**Group of diagnosis/symptoms (%)**				
Antenatal conditions	194 (20.1)	128 (19.7)	312 (43.7)	634 (27.2)
Respiratory	511 (52.9)	370 (56.8)	340 (47.6)	1221 (52.4)
Cardiovascular	39 (4.0)	29 (4.5)	29 (4.1)	97 (4.2)
Infectious	53 (5.5)	38 (5.8)	6 (0.8)	97 (4.2)
Hematologic	66 (6.8)	33 (5.1)	5 (0.7)	104 (4.5)
Other	103 (10.7)	53 (8.1)	22 (3.1)	178 (7.6)
**MINT**				
Mean (SD)	1.4 (3.5)	3.2 (5.5)	5.4 (5.7)	3.1 (5.2)
Median	0	0	4	0
**Birthplace (%)**				
Municipality of Rome	550 (56.9)	175 (26.9)	689 (96.5)	1414 (60.7)
Other	416 (43.1)	476 (73.1)	25 (3.5)	917 (39.3)
**Stabilization time (minutes)**				
Mean (SD)	31.6 (20.0)	39.6 (26.3)	28.5 (17.2)	32.9 (21.6)
Median	25	30	25	30
**TRIPS score pre**				
Mean (SD)	5.8 (7.4)	9.6 (10.8)	11.7 (11.3)	8.7 (10.0)
Median	5	6	6	6
**TRIPS change (%)**				
Deterioration	40 (4.1)	28 (4.3)	35 (4.9)	103 (4.4)
No change	697 (72.2)	445 (68.4)	557 (78.0)	1699 (72.9)
Improvement	229 (23.7)	178 (27.3)	122 (17.1)	529 (22.7)

^a^The Mortality Index for Neonatal Transportation was available for March 2010-December 2012.

## Conclusion

The findings suggest that specialized level of care contribute to improve the prognosis of sick infants, although the transportation may alter neonatal physiology. Future research may include also other process and outcomes measures.

## References

[B1] Powell-Tippit V Hertz D Stabilization and Preparation of the Infant for Transport Care of the Newborn: A Handbook for Primary Care 2005 Philadelphia, Lippincott, Williams & Wilkins 207 212

[B2] Teasdale D Hamilton C Baby on the move: issues in neonatal transport PaediatrNurs 2008 20 1 20 25 10.7748/paed2008.02.20.1.20.c6353 18335900

[B3] Goldsmit G Rabasa C Rodríguez S Aguirre Y Valdés M Pretz D Carmona D López Tornow S Fariña D Risk factors associated to clinical deterioration during the transport of sick newborn infants Arch Argent Pediatr 2012 110 4 304 309 22859323 10.5546/aap.2012.eng.304

[B4] Arora P Bajaj M Natarajan G Arora NP Kalra VK Zidan M Shankaran S Impact of interhospital transport on the physiologic status of very low-birth-weight infants Am J Perinatol 2014 31 3 237 244 23690051 10.1055/s-0033-1345259

[B5] Whitfield JM Buser MK Transport stabilization times for neonatal and pediatric patients prior to interfacility transfer PediatrEmerg Care 1993 9 2 69 71 10.1097/00006565-199304000-00002 8483782

[B6] Lee SK Zupancic JA Pendray M Thiessen P Schmidt B Whyte R Shorten D Stewart S Canadian Neonatal Network Transport risk index of physiologic stability: a practical system for assessing infant transport care J Pediatr 2001 139 2 220 226 10.1067/mpd.2001.115576 11487747

[B7] Broughton SJ Berry A Jacobe S Cheeseman P Tarnow-Mordi WO Greenough A Neonatal Intensive Care Unit Study Group The mortality index for neonatal transportation score: a new mortality prediction model for retrieved neonates Pediatrics 2004 114 4 e424 428 10.1542/peds.2003-0960-L 15466067

